# Glycerol Handling in Paired Visceral and Subcutaneous Adipose Tissues in Women with Normal Weight and Upper-Body Obesity

**DOI:** 10.3390/ijms25169008

**Published:** 2024-08-19

**Authors:** Anne Nørholm, Ida Guldbrandt Kjær, Esben Søndergaard, Birgitte Nellemann, Søren Nielsen, Janne Lebeck

**Affiliations:** 1 Department of Biomedicine, Aarhus University, 8200 Aarhus, Denmark; 201804742@post.au.dk (A.N.);; 2 Steno Diabetes Center Aarhus, Aarhus University Hospital, 8200 Aarhus, Denmark; esben.sondergaard@clin.au.dk (E.S.); soeren.nielsen@clin.au.dk (S.N.); 3 Center for Treatment of Rheumatic and Musculoskeletal Diseases (REMEDY), Diakonhjemmet Hospital, 0370 Oslo, Norway; 4 Department of Clinical Medicine, Aarhus University, 8000 Aarhus, Denmark

**Keywords:** aquaporin 7, cytosolic phosphoenolpyruvate carboxykinase, obesity, adipose tissue, lipolysis

## Abstract

In adipose tissue, reduced expression of the glycerol channel aquaporin 7 (AQP7) has been associated with increased accumulation of triglyceride. The present study determines the relative protein abundances of lipolytic enzymes, AQP7, and cytosolic phosphoenolpyruvate carboxykinase (PEPCK-C) in paired mesenteric and omental visceral adipose tissue (VAT) and abdominal and femoral subcutaneous adipose tissue (SAT) in women with either normal weight or upper-body obesity. No differences in the expression of hormone-sensitive lipase (HSL) or AQP7 were found between the two groups in the four depots. The expression of adipocyte triglyceride lipase (ATGL) and HSL were higher in omental VAT and femoral SAT than in mesenteric VAT in both groups of women. Similarly, AQP7 expression was higher in omental VAT than in mesenteric VAT. The expression of PEPCK-C was lower in omental VAT than in femoral SAT. No correlation between the expression of AQP7 and the mean adipocyte size was observed; however, the expression of PEPCK-C positively correlated with the mean adipocyte size. In conclusion, a depot-specific protein expression pattern was found for ATGL, HSL, AQP7, and PEPCK-C. The expression pattern supports that the regulation of AQP7 protein expression is at least in part linked to the lipolytic rate. Furthermore, the results support that the synthesis of glycerol-3-phosphate via glyceroneogenesis contributes to regulating triglyceride accumulation in white adipose tissue in women.

## 1. Introduction

During fasting, triglycerides (TG) stored in adipose tissue are hydrolyzed into free fatty acids (FFA) and glycerol by lipases. These lipases include adipose triglyceride lipase (ATGL), hormone sensitive lipase (HSL), and monoacylglycerol lipase. Even in the fasted state, a substantial part of the generated FFA is immediately re-esterified into TG within the adipocyte, i.e., before being released into the interstitial space of the adipose tissue [1]. The re-esterification requires glycerol-3-phosphate (G3P) that can be synthesized from glucose via glycolysis or from pyruvate via glyceroneogenesis, where cytosolic phosphoenolpyruvate carboxykinase (PEPCK-C) catalyzes the rate-limiting step. G3P can also be synthesized from glycerol after phosphorylation by glycerol kinase [1,2].

Aquaporin 7 (AQP7) is a glycerol channel which facilitates the efflux of glycerol from adipose tissue. White adipose tissues (WAT) only have minor glycerol kinase activity, and in general the fate of glycerol in adipose tissue is to be exported and metabolized in other tissues [2]. A study of AQP7 knockout mice has shown that AQP7 deficiency is associated with decreased efflux of glycerol, increased activity of glycerol kinase, and an increased accumulation of triglycerides in adipose tissue [3]. This suggests that AQP7 could be involved in the regulation of TG accumulation in adipose tissue by regulating the intra-adipocyte glycerol concentration. Supporting this, Prudente and coworkers found an association between a single nucleotide polymorphism (SNP) in the promoter region of AQP7 and obesity. The SNP resulted in a reduced expression of AQP7 and the association with obesity was found only in women [4]. Moreover, we have reported that female mice respond to 12 weeks of high-fat diet (HFD) with an increased protein expression of AQP7 in adipose tissue, whereas in male mice no increase was observed. The increased expression of AQP7 in female mice was paralleled by only male mice responding to the HFD with adipocyte hypertrophy [5], indicating that a high efflux of glycerol from adipose tissue is associated with reduced TG storage.

Many human studies investigating the expression of AQP7 in WAT in relation to obesity have analyzed mRNA levels in abdominal subcutaneous adipose tissue (abdominal SAT) and/or visceral adipose tissue (VAT) in mixed-gender cohorts. Most of these studies point towards obesity being associated with a reduced expression of AQP7 in abdominal SAT when compared with lean controls [4,6,7,8]. However, other studies report no differences in the expression of AQP7 between lean and obese individuals [9]. This includes our previous study of the protein expression of AQP7 in abdominal SAT in healthy lean men and men with obesity and type 2 diabetes [10]. In VAT, however, obesity has been associated with an increased expression of AQP7 [9], which could suggest a depot-specific regulation to reduce TG accumulation in VAT.

As indicated above, AQP7 expression in adipose tissue is influenced by sex [4,11,12]. For instance, women increase the protein expression of AQP7 in abdominal SAT in response to exercise training, while men decrease its expression [12]. Furthermore, studies in 3T3-L1 adipocytes [13] and ovariectomized mice supplemented with 17β-estradiol [14] have revealed that the promoter region of murine AQP7 contains an estrogen receptor response element and that estrogen induces the expression of AQP7 in VAT, but not SAT. Moreover, the high levels of follicle-stimulating hormone found in post-menopausal women has also been shown to reduce the expression of AQP7 in adipose tissue and thereby contribute to an increased accumulation of TG in WAT [15]. Overall, these results suggest that AQP7 expression in WAT influences TG storage in a depot- and sex-specific manner. In addition, it also indicates that AQP7 could be involved in determining the level of TG accumulation in different adipose tissue depots and thereby contribute to the sex-specific distribution pattern of fat in men and women, that generally protects premenopausal women against cardiometabolic disease [16].

We therefore hypothesized that (1) the expression of AQP7 protein in WAT from premenopausal women is related to obesity in a depot-specific manner, and (2) the relative expression of AQP7 protein is depot-specific and related to either lipolysis or TG storage in adipose tissue. Therefore, in this study we investigated whether the protein expression of HSL and AQP7 in WAT is different in women with upper-body obesity compared to women with normal weight in mesenteric and omental VAT and abdominal and femoral SAT. The four different WAT depots were included as they are central players in the sex-specific distribution pattern of fat [16]. We compared the protein expression of ATGL, HSL, AQP7, and PEPCK-C in the different depots and how their expression patterns are correlated. Finally, to evaluate the relationship between the expression of AQP7 and PEPCK-C and TG accumulation in adipocytes, we explored the relationship between the expression of AQP7 and PEPCK-C in WAT and the mean adipocyte size.

## 2. Results

The baseline characteristics are outlined in Table 1. All women were at their reproductive age. The mean sizes of the total upper-body and femoral SAT depots were greater in women with upper-body obesity compared to women with normal weight (by 80% and 42%, respectively) (Table 1). This was paralleled by a tendency towards a larger mean adipocyte size, especially in the SAT depots (Table 1). In lean women, the mean adipocyte size was larger in femoral SAT compared to omental VAT. This difference was also observed in women with upper-body obesity, where in addition the mean femoral SAT fat cell size was larger than the mean mesenteric fat cell size (Table 1).

### 2.1. HSL and AQP7 Expression in Women with Normal Weight or Upper-Body Obesity

**Figure 1:**
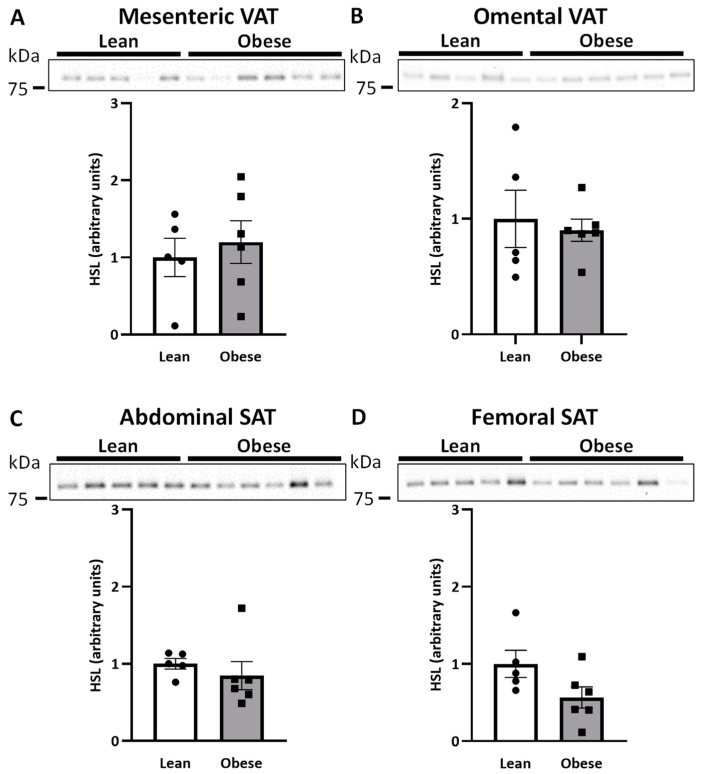
Relative quantification of hormone-sensitive lipase (HSL) in adipose tissue in women with normal weight (lean) (*n* = 5) and women with upper-body obesity (obese) (*n* = 6). Immunoblots and results of the densitometric analysis of the immunoblots are shown for HSL. Normalization was performed to the total amount of transferred protein in each lane (see Figure S1). (**A**). Relative HSL expression in mesenteric adipose tissue. (**B**). Relative HSL expression in omental adipose tissue. (**C**). Relative HSL expression in subcutaneous abdominal adipose tissue (Abdominal SAT) (**D**). Relative HSL expression in subcutaneous femoral adipose tissue (Femoral SAT). Values are expressed as mean ± SEM, and statistically significant differences between women with normal weight and women with upper-body obesity were evaluated using a non-parametric unpaired *t*-test (Mann-Whitney).

**Figure 2:**
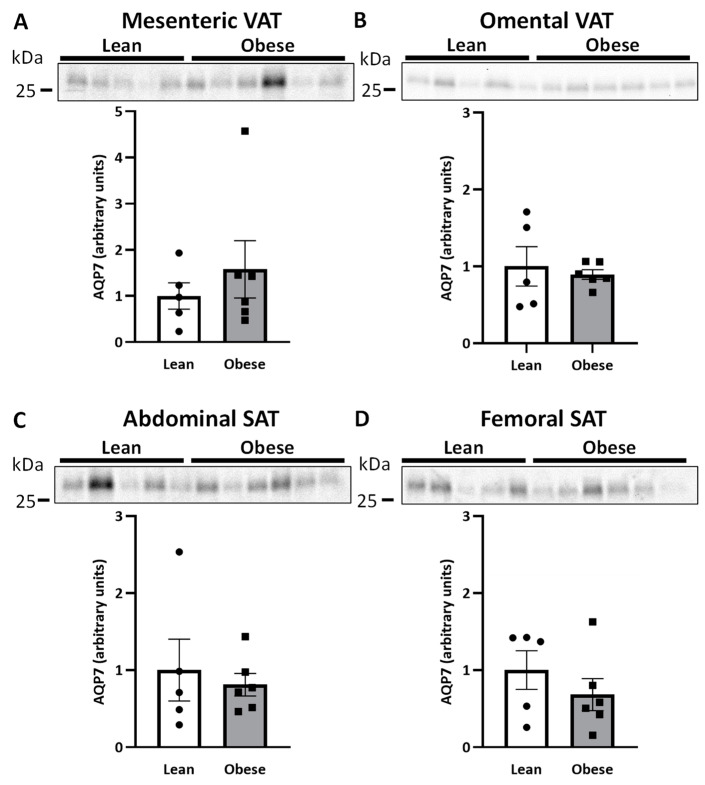
Relative quantification of aquaporin 7 (AQP7) in adipose tissue in women with normal weight (lean) (*n* = 5) and women with upper-body obesity (obese) (*n* = 6). Immunoblots and results of the densitometric analysis of the immunoblots are shown for AQP7. Normalization was performed to the total amount of transferred protein in each lane (see Figure S1). (**A**). Relative AQP7 expression in mesenteric adipose tissue. (**B**). Relative AQP7 expression in omental adipose tissue. (**C**). Relative AQP7 expression in subcutaneous abdominal adipose tissue (Abdominal SAT). (**D**). Relative AQP7 expression in subcutaneous femoral adipose tissue (Femoral SAT). Values are expressed as mean ± SEM, and statistically significant differences between women with normal weight and women with upper-body obesity were evaluated using a non-parametric unpaired *t*-test (Mann-Whitney).

We first wanted to compare the protein expression of HSL and AQP7 in women with normal weight and women with upper-body obesity in the different adipose tissue depots. No significant differences in the relative protein expression of HSL between the two groups were observed in mesenteric VAT, omental VAT, or SAT from the abdominal or femoral regions (Figure 1A–D), although a trend towards lower expression of HSL in femoral SAT was noted in women with upper-body obesity (*p* = 0.082). Similarly, no significant differences in the relative protein expression of AQP7 between the two groups were observed in any of the investigated depots (Figure 2A–D).

### 2.2. ATGL and HSL Expression in Paired Visceral and Subcutanous Adiposes Tissue Depots

**Figure 3:**
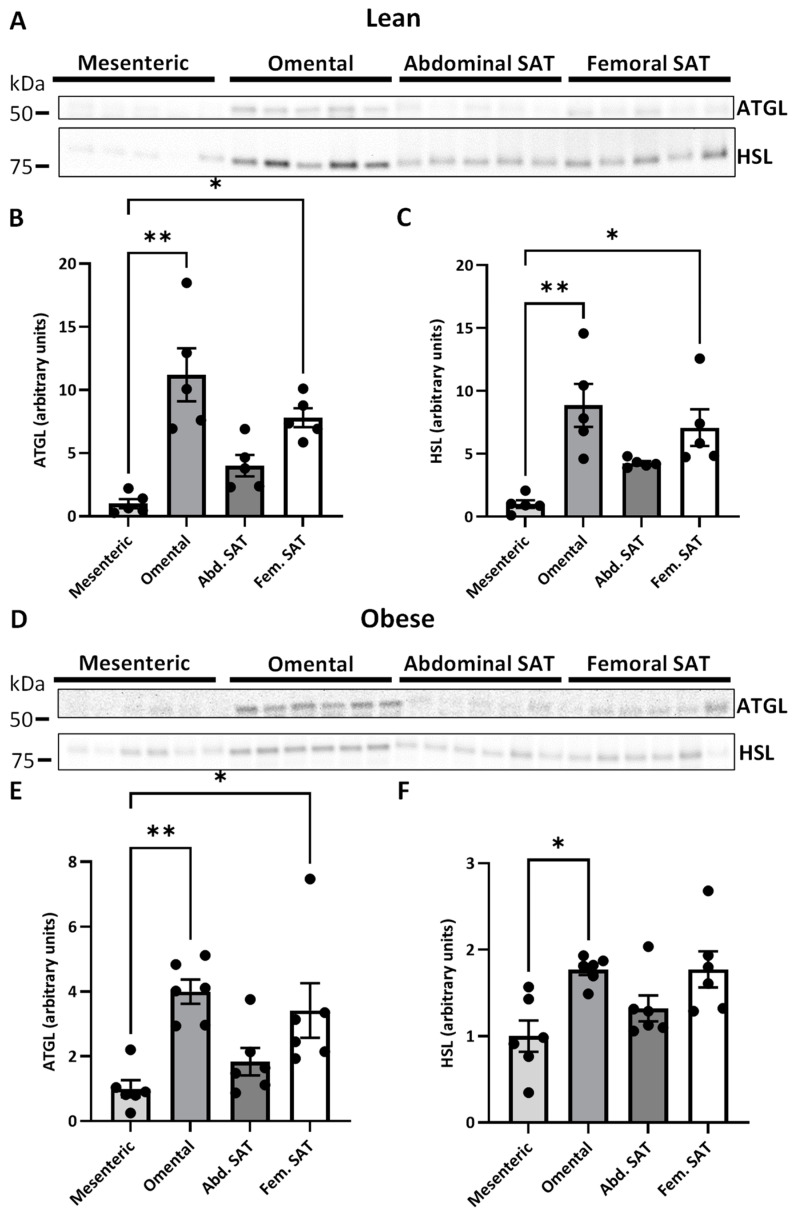
Relative quantification of adipose triglyceride lipase (ATGL) and hormone-sensitive lipase (HSL) in different adipose tissue depots in women with normal weight (lean) (*n* = 5) and women with upper-body obesity (obese) (*n* = 6). Immunoblots and results of the densitometric analysis of the immunoblots are shown for the indicated proteins. Normalization was performed to the total amount of transferred protein in each lane (see Figure S2). (**A**). Immunoblots for ATGL and HSL in mesenteric VAT, omental VAT, and SAT from the abdominal and femoral region in women with normal weight. (**B**). Relative ATGL expression in the indicated adipose tissue depots in women with normal weight. (**C**). Relative HSL expression in the indicated adipose tissue depots in women with normal weight. (**D**). Immunoblots for ATGL and HSL in mesenteric VAT, omental VAT, and SAT from the abdominal and femoral region of women with upper-body obesity (obese). (**E**) Relative ATGL expression in the indicated adipose tissue depots in women with upper-body obesity (obese). (**F**) Relative HSL expression in the indicated adipose tissue depots in women with upper-body obesity (obese). Statistically significant differences between the different adipose tissue depots were evaluated using a matched non-parametric one-way ANOVA (Friedman test). * Indicates statistical significance, with a *p*-value < 0.05, ** indicates a *p*-value < 0.01.

Next, we wanted to investigate the relative expression of ATGL and HSL in the different adipose tissue depots. In women with normal weight, omental VAT and femoral SAT had a higher mean protein abundance of both ATGL (11.2 ± 1.9 and 7.8 ± 0.7 vs. 1.0 ± 0.3, *p* = 0.009 and *p* = 0.020, respectively) (Figure 3B) and HSL (8.9 ± 1.6 and 7.1 ± 1.3 vs. 1.0 ± 0.3, *p* = 0.009 and *p* = 0.020, respectively) (Figure 3C) compared to mesenteric VAT. In women with upper-body obesity, a similar relative protein expression pattern was observed for ATGL (4.7 ± 0.4 and 2.3 ± 0.8 vs. 1.0 ± 0.3, *p* = 0.005 and *p* = 0.022, respectively) (Figure 3E). A trend towards a similar pattern was observed for HSL; however, a significant difference was only observed between omental and mesenteric VAT (1.7 ± 0.1 vs. 1.0 ± 0.2, *p* = 0.022) (Figure 3F).

### 2.3. AQP7 Expression in Paired Visceral and Subcutanous Adiposes Tissue Depots

**Figure 4:**
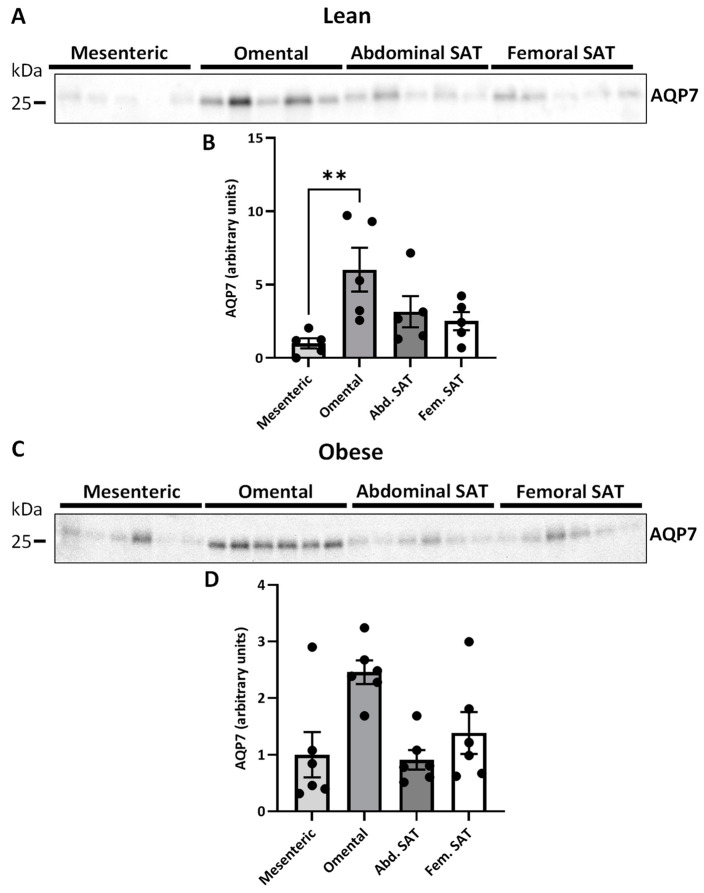
Relative quantification of the glycerol channel aquaporin 7 (AQP7) in different adipose tissue depots in women with normal weight (lean) (*n* = 5) and women with upper-body obesity (obese) (*n* = 6). Immunoblots and results of the densitometric analysis of the immunoblots are shown for the indicated proteins. Normalization was performed to the total amount of transferred protein in each lane (see Figure S3). (**A**). Immunoblot for AQP7 in mesenteric VAT, omental VAT, and SAT from the abdominal and femoral region of women with normal weight. (**B**). Relative AQP7 expression in the indicated adipose tissue depots in women with normal weight. (**C**). Immunoblot for AQP7 in the different adipose tissue depots in women with upper-body obesity. (**D**). Relative AQP7 expression in the indicated adipose tissue depots in women with upper-body obesity. Values are expressed as mean ± SEM, and statistically significant differences between the different adipose tissue depots were evaluated using a non-parametric matched one-way ANOVA (Friedman test). ** Indicates statistical significance, with a *p*-value < 0.01.

**Figure 5:**
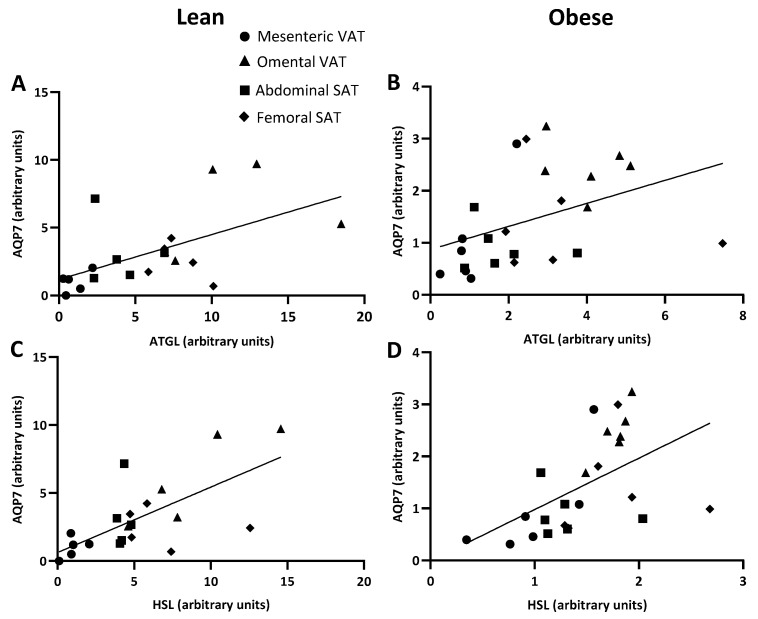
Relations between the protein expression of adipose triglyceride lipase (ATGL) or hormone sensitive lipase (HSL) and the glycerol channel aquaporin 7 (AQP7) in women with normal weight (lean) (*n* = 5) and women with upper-body obesity (obese) (*n* = 6). (**A**). Correlation between the protein expression of ATGL and AQP7 in the different adipose tissue depots (mesenteric: circles, omental: triangles, abdominal SAT: squares, femoral SAT: diamonds) in women with normal weight. (**B**). Correlation between the protein expression of ATGL and AQP7 in the different adipose tissue depots in women with upper-body obesity. (**C**). Correlation between the protein expression of HSL and AQP7 in the different adipose tissue depots in women with normal weight. (**D**). Correlation between the protein expression of HSL and AQP7 in different adipose tissue depots in women with upper-body obesity. Spearman’s rank correlation was used to determine whether a statistically significant correlation was found between the relative expressions of the indicated proteins.

Next, the expression of AQP7 protein was evaluated. In women with normal weight, the protein abundance of AQP7 was higher in omental VAT than in mesenteric VAT (6.0 ± 1.4 vs. 1.0 ± 0.3, *p* = 0.001). No significant differences were observed between abdominal or femoral SAT and mesenteric VAT (*p* = 0.300 and *p* = 0.519, respectively) (Figure 4B). In women with upper-body obesity, there were statistically significant differences in AQP7 expression between adipose tissue depots, as determined by non-parametric one-way ANOVA (*p* = 0.035). However, in the post hoc analysis the relative abundance of AQP7 only tended towards being higher in omental VAT than in mesenteric VAT (*p* = 0.083) and abdominal SAT (*p* = 0.083) (Figure 4D). When investigating the relationship between the expression of the two lipolytic enzymes and AQP7 across the different depots, a positive correlation was found between the protein expression of ATGL and AQP7 in both women with normal weight (*p* = 0.003, r = 0.630) and with upper-body obesity (*r* = 0.570, *p* = 0.004) (Figure 5A,B). Similarly, a positive correlation was found between the protein expression of HSL and AQP7 in both groups of women (*r* = 0.630, *p* = 0.003 and *r* = 0.647, *p* = 0.001, respectively) (Figure 5C,D).

### 2.4. PEPCK-C Expression in Paired Visceral and Subcutanous Adipose Tissue Depots

**Figure 6:**
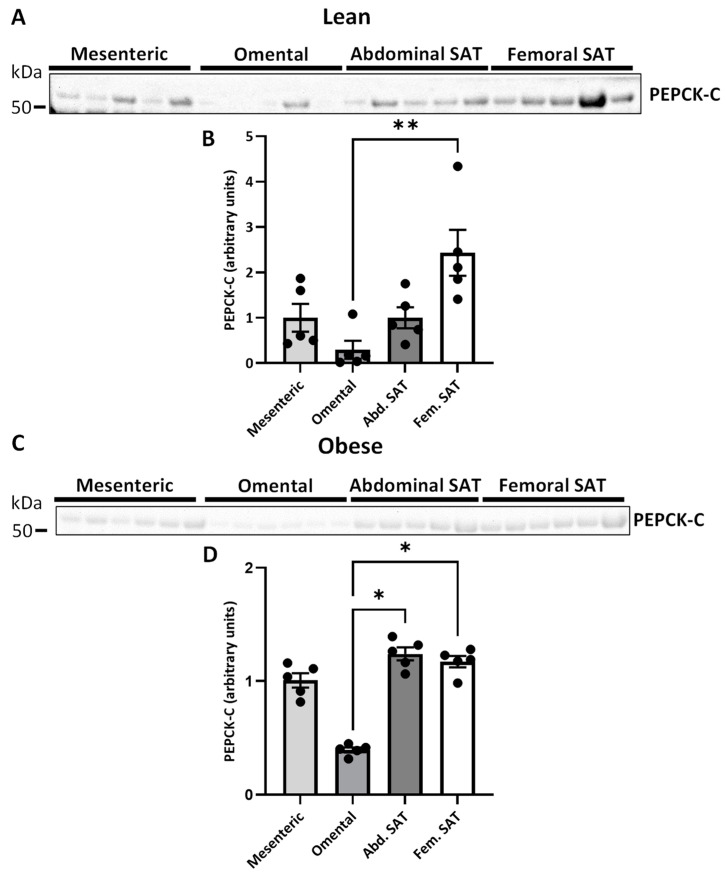
Relative quantification of cytosolic phosphoenolpyruvate carboxykinase (PEPCK-C) in different adipose tissue depots in women with normal weight (lean) (*n* = 5) and women with upper-body obesity (obese) (*n* = 5–6). Immunoblots and results of the densitometric analysis of the immunoblots are shown for the indicated proteins. Normalization was performed to the total amount of transferred protein in each lane (see Figure S4). (**A**). Immunoblot for PEPCK-C in mesenteric VAT, omental VAT, and SAT from the abdominal and femoral regions of women with normal weight. (**B**). Relative PEPCK-C expression in the indicated adipose tissue depots. (**C**). Immunoblot for PEPCK-C in the different adipose tissue depots in women with upper-body obesity (*n* = 5–6). (**D**). Relative PEPCK-C expression in the indicated adipose tissue depots in women with upper-body obesity. To be able to perform a paired statistical analysis, only 5 of the 6 individuals were included in the analysis. Values are expressed as mean ± SEM, and statistically significant differences between the different adipose tissue depots were evaluated using a non-parametric matched one-way ANOVA (Friedman test). * Indicates statistical significance, with a *p*-value < 0.05, ** indicates a *p*-value < 0.01.

**Figure 7:**
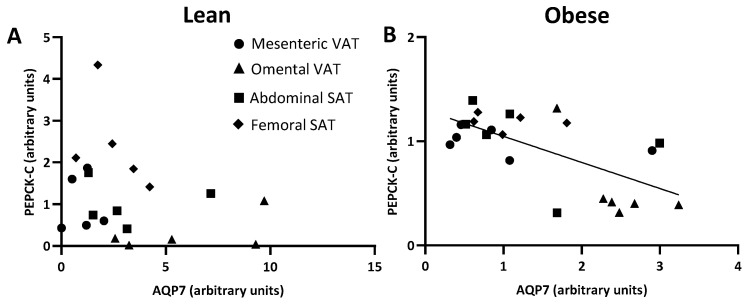
Relations between the protein expression of the glycerol channel aquaporin 7 (AQP7) and cytosolic phosphoenolpyruvate carboxykinase (PEPCK-C) in women with normal weight (lean) (*n* = 5) and women with upper-body obesity (obese) (*n* = 5–6). (**A**). Correlation between the protein expression of AQP7 and PEPCK-C in the different adipose tissue depots (mesenteric: circles, omental: triangles, abdominal SAT: squares, femoral SAT: diamonds) in women with normal weight. (**B**). Correlation between the protein expression of AQP7 and PEPCK-C in the different adipose tissue depots in women with upper-body obesity (obese). Spearman’s rank correlation was used to determine whether a statistically significant correlation was found between the relative expressions of the indicated proteins.

In women with normal weight, omental VAT had a lower protein expression of PEPCK-C than femoral SAT (0.3 ± 0.2 vs. 2.4 ± 0.5, *p* = 0.009) (Figure 6B). A similar pattern was observed in women with upper-body obesity where the relative protein expression of PEPCK-C in omental VAT was lower than in both abdominal and femoral SAT (0.4 ± 0.0 vs. 1.2 ± 0.1 and 1.2 ± 0.1, *p* = 0.020 and *p* = 0.020, respectively) (Figure 6D). When investigating the relationship between the expression of AQP7 and PEPCK-C across depots, no significant correlation was found in women with normal weight (*p* = 0.169) (Figure 7A). By contrast, a negative correlation between the adipose tissue expression of AQP7 and PEPCK-C was found in women with upper-body obesity (*r* = −0.518, *p* = 0.011) (Figure 7B).

### 2.5. AQP7 and PEPCK-C Expression and Mean Fat Cell Size

**Figure 8:**
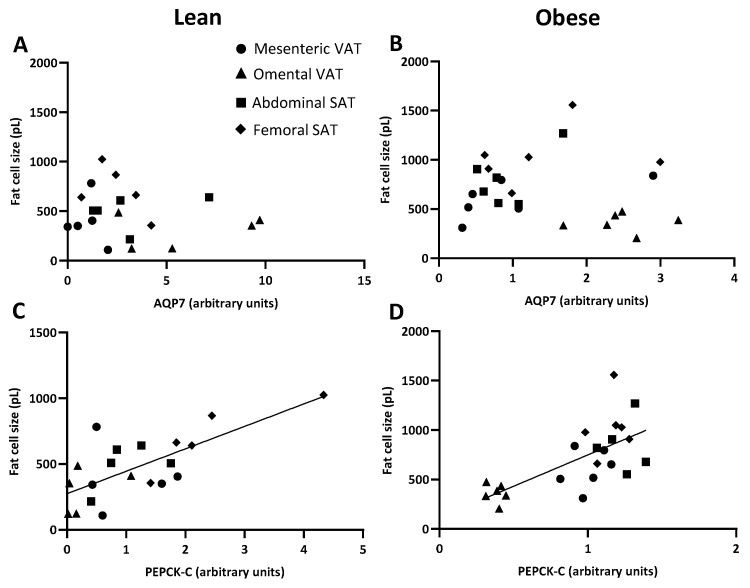
Relations between the protein expression of glycerol channel aquaporin 7 (AQP7) or cytosolic phosphoenolpyruvate carboxykinase (PEPCK-C) and mean fat cell size (pL) in women with normal weight (lean) (*n* = 5) and women with upper-body obesity (obese) (*n* = 5–6). (**A**). Correlation between the protein expression of AQP7 and the mean adipocyte size in the different adipose tissue depots (mesenteric: circles, omental: triangles, abdominal SAT: squares, femoral SAT: diamonds) in women with normal weight. (**B**). Correlation between the protein expression of AQP7 and the mean adipocyte size in the different adipose tissue depots in women with upper-body obesity. (**C**). Correlation between the protein expression of PEPCK-C and the mean adipocyte size in the different adipose tissue depots in women with normal weight. (**D**). Correlation between the protein expression of PEPCK-C and the mean adipocyte size in the different adipose tissue depots in women with upper-body obesity. Spearman’s rank correlation was used to determine whether a statistically significant correlation was found between the relative expressions of the indicated proteins and the mean adipocyte size.

Next, we wanted to investigate whether the protein expressions of AQP7 and PEPCK-C with their influence on the availability of G3P for TG synthesis correlate with the mean size of the adipocytes across adipose tissue depots. No correlation between the abundance of AQP7 protein and mean fat cell size across the different depots was found in either women with normal weight (*p* = 0.548) or women with upper-body obesity (*p* = 0.557) (Figure 8A,B). For PEPCK-C protein abundance, a positive correlation with the mean fat cell size was observed in both lean (*r* = 0.657, *p* = 0.002) and upper-body obese women (*r* = 0.739, *p* < 0.001) (Figure 8C,D).

## 3. Discussion

In the present study, we measured the relative protein abundances of ATGL, HSL, AQP7, and PEPCK-C in mesenteric and omental VAT and abdominal and femoral SAT depots in overnight fasted women with either normal weight or upper-body obesity. First, the protein expressions of both HSL and AQP7 did not differ between the two groups of women in any of the investigated WAT depots. Second, the relative protein abundances of ATGL, HSL, AQP7, and PEPCK-C all differed between depots in both groups of women. Third, across the different WAT depots, the expression of AQP7 protein positively correlated with the expression of ATGL and HSL, whereas a negative correlation with the protein expression of PEPCK-C was found in women with upper-body obesity. Fourth, the protein expression of PEPCK-C but not AQP7 correlated with the mean fat cell size.

The results do not support that obesity is associated with marked changes in the protein expression of HSL and AQP7 in WAT or that there is a depot-specific effect of being obese on the expression of AQP7 in WAT when comparing women with upper-body obesity with lean women.

In abdominal SAT, a strong correlation between the protein expression of HSL and HSL activity has been described [17]. Thus, the lack of a significant difference in the relative protein abundance of HSL in the different adipose tissue depots between women with normal weight and women with upper-body obesity points towards no significant differences in the adipose tissue lipolytic rate between the two groups of women. Previous studies of the protein expression of HSL in individuals with obesity have reported a reduced expression in abdominal SAT from men and women with obesity [18,19]. However, the results for HSL were made in individuals with a higher BMI than the BMI of the women included in this study (mean BMI > 40 kg/m^2^ [17] or with a BMI ranging from 30–52 kg/m^2^ [19]).

In contrast to the findings in this study, some studies have found that obesity is associated with a reduced expression of AQP7 in abdominal SAT. Several factors could contribute to this discrepancy. First of all, this study only investigated women at their reproductive age. Furthermore, the majority of previous studies have investigated mRNA levels [4,6,7], whereas the present study analyzed the relative protein expression of AQP7. Similar to HSL, the reduced AQP7 expression in abdominal SAT reported in other studies was performed in individuals with obesity that generally had a higher BMI (35 kg/m^2^ [6] or >40 kg/m^2^ [4,7,8]) than the mean BMI of the women included in this study. As with our results, no significant changes in the expression of AQP7 mRNA have been reported in abdominal SAT in individuals with obesity with a mean BMI < 35 kg/m^2^ [7,9,10]. Overall, this suggests, that a reduced expression of HSL and AQP7 in abdominal SAT is mainly found in morbidly obese individuals.

Previous studies have shown a strong correlation between the expression of ATGL and HSL in different adipose tissue depots in both lean and upper-body obese women [20,21,22], indicating that the two lipolytic enzymes share regulatory mechanisms. With AQP7 facilitating the release of glycerol derived from lipolysis, we expected the protein expression of AQP7 to correlate with the expression of ATGL and HSL. The positive correlations between the protein expressions of AQP7 and the two lipolytic enzymes support that the expression of AQP7 in humans is at least partly linked to the overall lipolytic rate in adipose tissue depots. This is in line with the study by Mirinda and coworkers, where the mRNA expression of AQP7 and ATGL / HSL were positively correlated in both abdominal SAT and VAT [9]. In a previous study, we found no linear association between the protein expression of AQP7 in abdominal SAT in lean and obese men, using palmitate flux as a marker of the lipolytic rate in adipose tissue [10]. Similarly, in mice the protein expression of AQP7 was only significantly increased after 72 h of fasting, whereas after 24 h no difference in expression was observed [23] despite that 24 h of fasting in mice would be associated with a high lipolytic rate. Overall, the present results support that in premenopausal women the total AQP7 protein expression is at least partially linked to the regulatory mechanisms that promotes lipolysis in adipose tissue depots and that a high lipolytic rate is associated with an increased efflux of glycerol via AQP7 in WAT.

Glyceroneogensis is an important pathway for synthesizing G3P in adipose tissue, and studies in mice have shown that adipose tissue-specific overexpression of PEPCK results in increased adipocyte size and fat mass, and higher body weight [24]. In this study, the relative protein abundance of PEPCK-C was similar to the expression of lipolytic enzymes and AQP7 depot-specific in both groups of women. We found a low expression of PEPCK-C in omental VAT compared to femoral SAT in women with normal weight, and in the women with upper-body obesity, the expression of PEPCK-C in omental VAT was lower compared to both abdominal and femoral SAT. In all, the results suggest that both a relatively higher lipolytic rate and efflux of glycerol via AQP7 and a lower generation of G3P via glyceroneogenesis together protect the omental VAT against exaggerated TG accumulation in premenopausal women. In addition, it also highlights the importance of depot- and sex-specific investigations when evaluating adipose tissue biology.

Moreover, the expression of AQP7 correlated with PEPCK-C in women with upper-body obesity, suggesting an inverse relationship between the expression of AQP7 and PEPCK-C, especially in omental VAT.

Finally, the lack of correlation between the relative protein expression of AQP7 in the different depots and their mean adipocyte size suggests that the expression of AQP7 is not a strong determinant of adipocyte size. To our knowledge, this is the first analysis of the relationship between AQP7 expression and mean adipocyte size in a human setting. The results contradict that a low expression of AQP7 in adipose tissue per se should promote adipocyte hypertrophy. Instead, we found a positive correlation between the relative protein expression of PEPCK-C in the different depots and the mean adipocyte size in both women with normal weight and women with upper-body obesity, thus supporting that synthesis of G3P via glyceroneogenesis contributes to the regulation of TG accumulation in WAT in humans.

This study has some limitations. First, the small number of individuals investigated increases the risk of not identifying differences, especially with the rather large inter-individual variation in the expression of AQP7 and PEPCK-C proteins in adipose tissue. Second, we only investigated the total protein abundance of ATGL and HSL and not the activity of the enzymes or the phosphorylation status of the proteins. Furthermore, in adipose tissue, AQP7 is expressed both in the adipocytes and the capillary endothelial cells [25] and in adipocytes, there is evidence for trafficking of AQP7 from intracellular domains to the plasma membrane domain in response to epinephrine/isoproterenol stimulation [8,26,27,28]. In this study, we did not investigate the cellular or subcellular localization of AQP7 in the investigated adipose tissues, and further studies are needed to determine the relationship between the cellular and subcellular expression of AQP7 in adipose tissue and obesity.

## 4. Materials and Methods

### 4.1. Adipose Tissue Samples

The samples used for immunoblotting were adipose tissue biopsies collected in a previous study [29]. As previously described [29], adipose tissue samples were obtained from women with upper-body obesity (waist circumference > 88 cm) and lean women (waist circumference < 80 cm) listed for voluntary laparoscopic tubal occlusion. The women were healthy non-smokers and used no medications except oral contraceptives.

The local ethics committee had approved the protocol (VEK: M2009-0057) and informed consent was obtained from all women. Adipose tissue biopsies were collected as previously described from (1) the periumbilical region (abdominal SAT), (2) the anterior femoral region (femoral SAT), and (3) from visceral depots (mesenteric and omental VAT) after a 10–14 h overnight fast and stored at −80 °C until used. Dual-energy X-ray absorptiometry scan and abdominal computed tomography scan at the L2–L3 interval were performed to obtain anthropometric measures. Fat cell size was determined immediately after collection of the biopsy as previously described [30]. In the present study, we included biopsies from 5 lean women and 6 women with upper-body obesity.

### 4.2. Semiquantitative Immunoblotting

*Sample preparation*: Adipose tissue was homogenized in ice cold dissection buffer (0.3 M sucrose, 25 mM imidazole, 1 mM EDTA in MilliQ H_2_O, pH 7.2 containing 8.4 μM leupeptin, and 0.4 mM Pefabloc). The homogenate was centrifuged at 4000 × *g* for 15 min at 4 °C. Supernatants were mixed with sample buffer, giving a final concentration of 62 mM Tris, 0.1 M sodium dodecyl sulfate, 8.7% glycerol, 0.09 mM bromophenol blue, and 0.04 M DTT, pH 6.8, heated for 15 min at 65 °C and stored at −20 °C until use. Protein separation was performed using Any kD Criterion TGX precast protein gels (Bio-Rad Laboratories, Copenhagen, Denmark), and protein loading was adjusted to ensure equal loading (± 10%) using Gelcode Coomassie blue stain reagent (Thermo Fisher Scientific, Waltham, MA, USA).

*Immunoblotting*: Protein separation was performed using Any kD Criterion TGX precast protein gels (Bio-Rad Laboratories, Copenhagen, Denmark), and separated proteins were electro-transferred to PVDF membranes using the Trans-Blot Turbo transfer system (Bio-Rad Laboratories, Copenhagen, Denmark). After transfer, the total amount of transferred protein in each lane was detected using the No-Stain Protein Labeling Reagent (Invitrogen, Waltham, MA, USA) according to the manufacturer’s protocol (Figure S1–S4). The signal was detected using the *iBright CL1500* Imaging System (Thermo Fisher Scientific, Waltham, MA, USA). The membranes were then blocked for one hour in PBS-T with 5% milk. The membranes were washed and then cut into parts, and each part was incubated overnight with one primary antibody against either HSL (#4107, Cell Signaling Technology, Danvers, MA, USA), human AQP7 (8571) [11], ATGL (#2138, Cell Signaling Technology, Danvers, MA, USA), or PEPCK-C (#10004943, Cayman Chemical Ann Arbor, MI, USA) in PBS-T with 1% bovine serum albumin and 2mM NaN_3_. After washing, the membranes were incubated with appropriate HRP-conjugated secondary antibodies. After a washing step, ECL Western Blotting Substrate (Pierce™, Waltham, MA, USA) was added, and chemiluminescence was detected by Image-Quant Las4000. ImageJ software was used to subtract background and quantify band intensities. Band intensities were adjusted to the amount of transferred protein in each lane, detected from the same membrane by the No-Stain Protein Labeling Reagent (Invitrogen, Waltham, MA, USA). This normalization approach was chosen to avoid a potential differential expression of standard reference proteins between lean and obese individuals or the different adipose tissue depots investigated [31].

### 4.3. Presentation of Data and Statistical Analyses

Data are presented as mean ± SEM. Statistical analysis was accomplished using a non-parametric unpaired Student’s *t*-test (Mann-Witney) when comparing lean women and women with upper-body obesity. For comparison of the adipose tissue depots, a non-parametric one-way repeated measures ANOVA (Friedman) was used. The association between protein abundances or protein abundance and mean fat cell size or the adipose tissue depot size were evaluated with Spearman rank-order correlation. The statistical tests were performed using GraphPad Prism 10.2.3; *p*-values < 0.05 were considered statistically significant.

## 5. Conclusions

In conclusion, we found no evidence for overnight fasted premenopausal women with upper-body obesity with a BMI ranging from 28–37 kg/m^2^ having abnormal protein expression of HSL and AQP7 in mesenteric and omental VAT or abdominal and femoral SAT when compared to normal weight controls. Interestingly, we found that the relative protein expression of ATGL, HSL, and AQP7 was depot-specific and positively correlated across depots. These results support that the regulation of AQP7 protein is at least in part linked to the lipolytic rate. Additionally, the expression of PEPCK-C was also depot-specific. Taken together, the depot-specific expression of lipolytic enzymes, AQP7, and PEPCK-C could promote a lower accumulation of TG in omental VAT in the investigated women. Finally, we found no evidence for an inverse relationship between the expression of AQP7 protein and the mean adipocyte size. Instead, the present data support that synthesis of G3P via glyceroneogenesis contributes to regulating TG accumulation in WAT in women.

## Figures and Tables

**Table 1:** Clinical characteristics of the participants. The values are presented as mean (range) or mean ± SEM, and statistically significant differences between women with normal weight (lean) and women with upper-body obesity (obese) were evaluated using a non-parametric unpaired *t*-test (Mann-Whitney).

	Lean	Obese	*p*-Value
*n*	5	6	
Age (years)	39 (33–43)	35 (28–40)	0.301
BMI (kg/m^2^)	23 (22–25)	32 (28–37) *	0.004
Total VAT (g)	1071 ± 258	2052 ± 298	0.052
Total upper-body SAT (g)	8904 ± 978	16,007 ± 874 *	0.004
Total femoral SAT (g)	10,237 ± 638	14,579 ± 692 *	0.004
Mesenteric fat cell size (pL)	398 ± 109	603 ± 81	0.157
Omental fat cell size (pL)	299 ± 75	363 ± 39	0.792
Abdominal fat cell size (pL)	496 ± 75	798 ± 110	0.059
Femoral fat cell size (pL)	710 ± 113 ^a^	1030 ± 120 ^a,b^	0.089

* Indicates statistical significance when compared to women with normal weight with a *p*-value < 0.05. Comparison of the mean fat cell size (pL) between depots was performed using a non-parametric one-way repeated measures ANOVA (Friedman). ^a^ Indicates statistical significance from omental VAT in the same group, ^b^ Indicates statistical significance from mesenteric VAT in the same group with a *p*-value < 0.05.

## Data Availability

The data presented in this study are available on request from the corresponding author.

## Supplementary Materials

The following supporting information can be downloaded at: https://www.mdpi.com/article/10.3390/ijms25169008/s1.

## References

[1] Reshef L., Olswang Y., Cassuto H., Blum B., Croniger C.M., Kalhan S.C., Tilghman S.M., Hanson R.W. (2003). Glyceroneogenesis and the triglyceride/fatty acid cycle. J. Biol. Chem..

[2] Xue L.L., Chen H.H., Jiang J.G. (2017). Implications of glycerol metabolism for lipid production. Prog. Lipid Res..

[3] Hibuse T., Maeda N., Funahashi T., Yamamoto K., Nagasawa A., Mizunoya W., Kishida K., Inoue K., Kuriyama H., Nakamura T. (2005). Aquaporin 7 deficiency is associated with development of obesity through activation of adipose glycerol kinase. Proc. Natl. Acad. Sci. USA.

[4] Prudente S., Flex E., Morini E., Turchi F., Capponi D., De Cosmo S., Tassi V., Guida V., Avogaro A., Folli F. (2007). A functional variant of the adipocyte glycerol channel aquaporin 7 gene is associated with obesity and related metabolic abnormalities. Diabetes.

[5] Iena F.M., Jul J.B., Vegger J.B., Lodberg A., Thomsen J.S., Bruel A., Lebeck J. (2020). Sex-Specific Effect of High-Fat Diet on Glycerol Metabolism in Murine Adipose Tissue and Liver. Front. Endocrinol..

[6] Marrades M.P., Milagro F.I., Martinez J.A., Moreno-Aliaga M.J. (2006). Differential expression of aquaporin 7 in adipose tissue of lean and obese high fat consumers. Biochem. Biophys. Res. Commun..

[7] Ceperuelo-Mallafre V., Miranda M., Chacon M.R., Vilarrasa N., Megia A., Gutierrez C., Fernandez-Real J.M., Gomez J.M., Caubet E., Fruhbeck G. (2007). Adipose tissue expression of the glycerol channel aquaporin-7 gene is altered in severe obesity but not in type 2 diabetes. J. Clin. Endocrinol. Metab..

[8] Rodriguez A., Catalan V., Gomez-Ambrosi J., Garcia-Navarro S., Rotellar F., Valenti V., Silva C., Gil M.J., Salvador J., Burrell M.A. (2011). Insulin- and Leptin-Mediated Control of Aquaglyceroporins in Human Adipocytes and Hepatocytes Is Mediated via the PI3K/Akt/mTOR Signaling Cascade. J. Clin. Endocrinol. Metab..

[9] Miranda M., Escote X., Ceperuelo-Mallafre V., Alcaide M.J., Simon I., Vilarrasa N., Wabitsch M., Vendrell J. (2010). Paired subcutaneous and visceral adipose tissue aquaporin-7 expression in human obesity and type 2 diabetes: Differences and similarities between depots. J. Clin. Endocrinol. Metab..

[10] Lebeck J., Sondergaard E., Nielsen S. (2018). Increased AQP7 abundance in skeletal muscle from obese men with type 2 diabetes. Am. J. Physiol.-Endocrinol. Metab..

[11] Sjoholm K., Palming J., Olofsson L.E., Gummesson A., Svensson P.A., Lystig T.C., Jennische E., Brandberg J., Torgerson J.S., Carlsson B. (2005). A Microarray search for genes predominantly expressed in human omental adipocytes: Adipose tissue as a major production site of serum amyloid A. J. Clin. Endocrinol. Metab..

[12] Lebeck J., Ostergard T., Rojek A., Fuchtbauer E.M., Lund S., Nielsen S., Praetorius J. (2012). Gender-specific effect of physical training on AQP7 protein expression in human adipose tissue. Acta Diabetol..

[13] Xing L., Jin B., Fu X., Zhu J., Guo X., Xu W., Mou X., Wang Z., Jiang F., Zhou Y. (2019). Identification of functional estrogen response elements in glycerol channel Aquaporin-7 gene. Climacteric.

[14] Jin B., Chen X., Xing L., Xu W., Fu X., Zhu J., Mou X., Wang Z., Shu J. (2017). Tissue-specific effects of estrogen on glycerol channel aquaporin 7 expression in an ovariectomized mouse model of menopause. Climacteric.

[15] Chen L., Chen H., Liu X., Li J., Gao Q., Shi S., Wang T., Ye X., Lu Y., Zhang D. (2020). AQP7 mediates post-menopausal lipogenesis in adipocytes through FSH-induced transcriptional crosstalk with AP-1 sites. Reprod. Biomed. Online.

[16] Mauvais-Jarvis F. (2024). Sex differences in energy metabolism: Natural selection, mechanisms and consequences. Nat. Rev. Nephrol..

[17] Large V., Arner P., Reynisdottir S., Grober J., Van Harmelen V., Holm C., Langin D. (1998). Hormone-sensitive lipase expression and activity in relation to lipolysis in human fat cells. J. Lipid Res..

[18] Large V., Reynisdottir S., Langin D., Fredby K., Klannemark M., Holm C., Arner P. (1999). Decreased expression and function of adipocyte hormone-sensitive lipase in subcutaneous fat cells of obese subjects. J. Lipid Res..

[19] Lofgren P., Hoffstedt J., Ryden M., Thorne A., Holm C., Wahrenberg H., Arner P. (2002). Major gender differences in the lipolytic capacity of abdominal subcutaneous fat cells in obesity observed before and after long-term weight reduction. J. Clin. Endocrinol. Metab..

[20] Steinberg G.R., Kemp B.E., Watt M.J. (2007). Adipocyte triglyceride lipase expression in human obesity. Am. J. Physiol. Endocrinol. Metab..

[21] Jocken J.W., Langin D., Smit E., Saris W.H., Valle C., Hul G.B., Holm C., Arner P., Blaak E.E. (2007). Adipose triglyceride lipase and hormone-sensitive lipase protein expression is decreased in the obese insulin-resistant state. J. Clin. Endocrinol. Metab..

[22] Mairal A., Langin D., Arner P., Hoffstedt J. (2006). Human adipose triglyceride lipase (PNPLA2) is not regulated by obesity and exhibits low in vitro triglyceride hydrolase activity. Diabetologia.

[23] Skowronski M.T., Lebeck J., Rojek A., Praetorius J., Fuchtbauer E.M., Frokiaer J., Nielsen S. (2007). AQP7 is localized in capillaries of adipose tissue, cardiac and striated muscle: Implications in glycerol metabolism. Am. J. Physiol.-Ren. Physiol..

[24] Franckhauser S., Munoz S., Pujol A., Casellas A., Riu E., Otaegui P., Su B., Bosch F. (2002). Increased fatty acid re-esterification by PEPCK overexpression in adipose tissue leads to obesity without insulin resistance. Diabetes.

[25] Iena F.M., Kalucka J., Nielsen L., Sondergaard E., Nielsen S., Lebeck J. (2022). Localization of aquaglyceroporins in human and murine white adipose tissue. Histochem. Cell Biol..

[26] Kishida K., Kuriyama H., Funahashi T., Shimomura I., Kihara S., Ouchi N., Nishida M., Nishizawa H., Matsuda M., Takahashi M. (2000). Aquaporin adipose, a putative glycerol channel in adipocytes. J. Biol. Chem..

[27] Hansen J.S., Krintel C., Hernebring M., Haataja T.J.K., de Mare S., Wasserstrom S., Kosinska-Eriksson U., Palmgren M., Holm C., Stenkula K.G. (2016). Perilipin 1 binds to aquaporin 7 in human adipocytes and controls its mobility via protein kinase A mediated phosphorylation. Metab. Clin. Exp..

[28] Laforenza U., Scaffino M.F., Gastaldi G. (2013). Aquaporin-10 Represents an Alternative Pathway for Glycerol Efflux from Human Adipocytes. PLoS ONE.

[29] Sondergaard E., Nellemann B., Sorensen L.P., Gormsen L.C., Christiansen J.S., Ernst E., Dueholm M., Nielsen S. (2011). Similar VLDL-TG storage in visceral and subcutaneous fat in obese and lean women. Diabetes.

[30] Di Girolamo M., Mendlinger S., Fertig J.W. (1971). A simple method to determine fat cell size and number in four mammalian species. Am. J. Physiol..

[31] Perez-Perez R., Lopez J.A., Garcia-Santos E., Camafeita E., Gomez-Serrano M., Ortega-Delgado F.J., Ricart W., Fernandez-Real J.M., Peral B. (2012). Uncovering suitable reference proteins for expression studies in human adipose tissue with relevance to obesity. PLoS ONE.

