# Supplementary material for: Glycerol Handling in Paired Visceral and Subcutaneous Adipose Tissues in Women with Normal Weight and Upper-Body Obesity
Source: Int J Mol Sci. 2024 Aug 19;25(16):9008. doi: 10.3390/ijms25169008 (PMC11354935; doi:10.3390/ijms25169008)
Supplement: Supplementary file 1 [file ijms-25-09008-s001.zip › ijms-3117300-supplementary.pptx]

## Slide 1
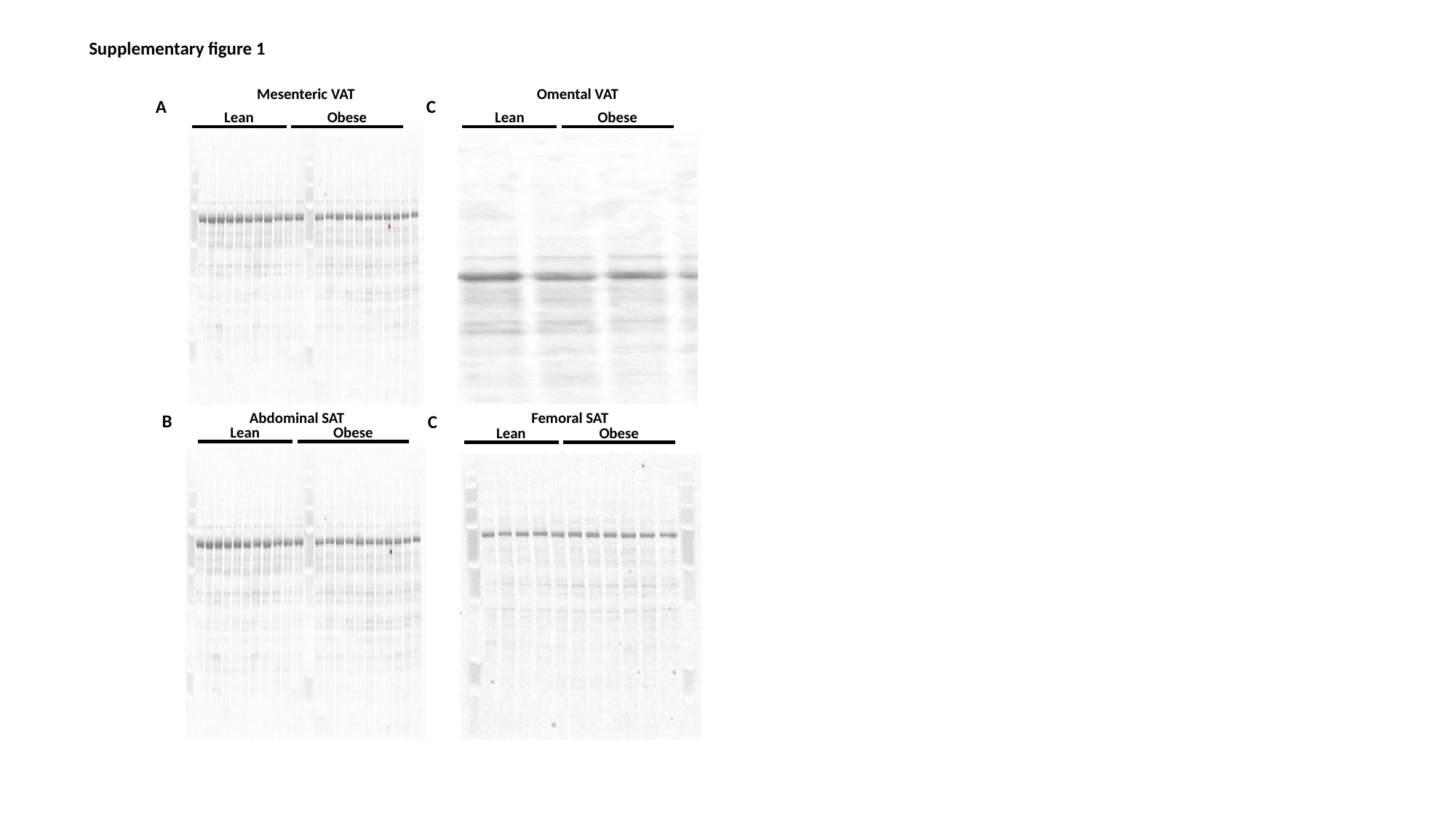

Supplementary figure 1
Mesenteric VAT
Omental VAT
A
C
Lean
Obese
Lean
Obese
Abdominal SAT
Femoral SAT
B
C
Lean
Obese
Lean
Obese

## Slide 2
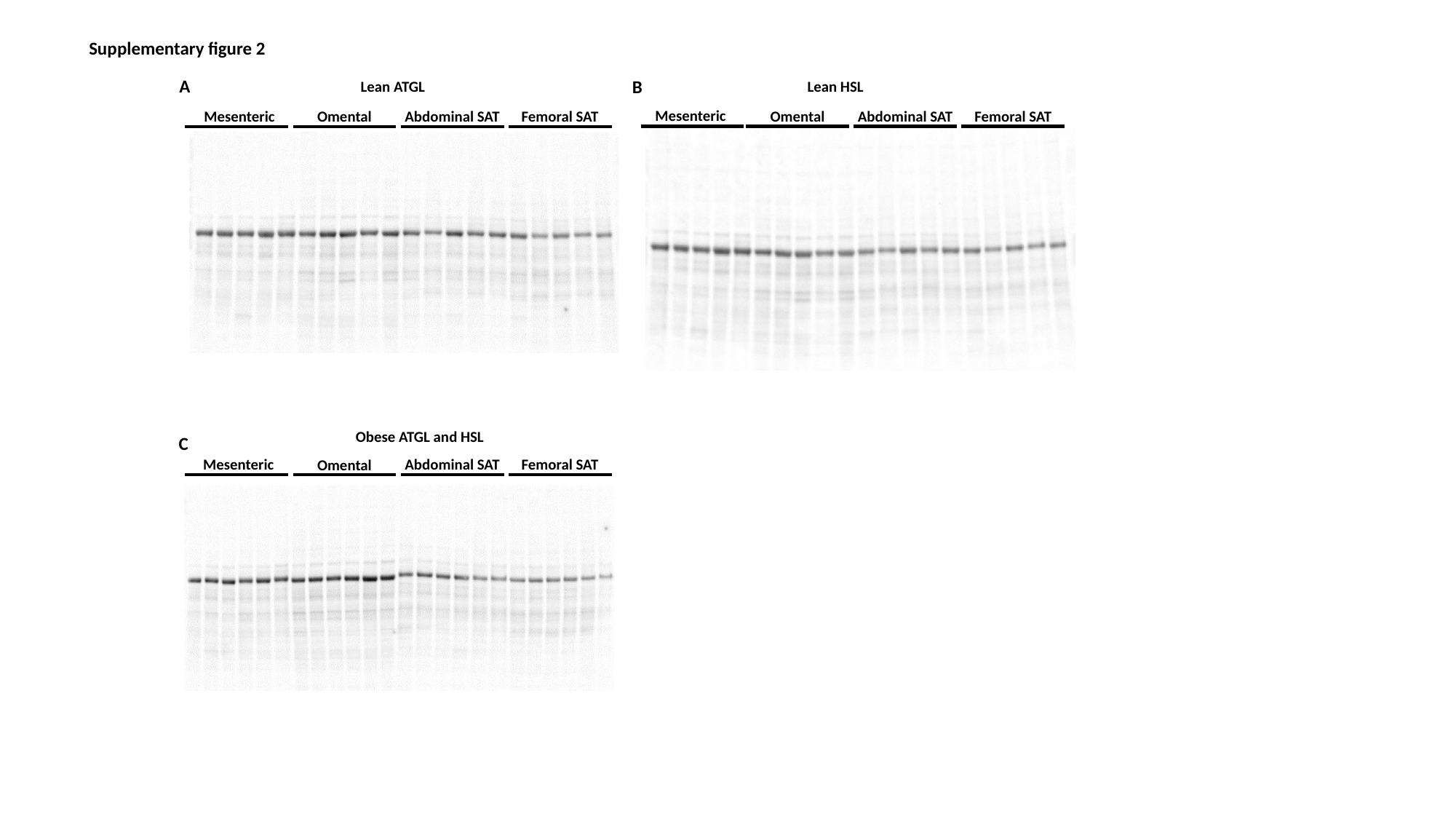

Supplementary figure 2
A
B
Lean HSL
Lean ATGL
Mesenteric
Abdominal SAT
Femoral SAT
Omental
Mesenteric
Abdominal SAT
Femoral SAT
Omental
Obese ATGL and HSL
C
Mesenteric
Abdominal SAT
Femoral SAT
Omental

## Slide 3
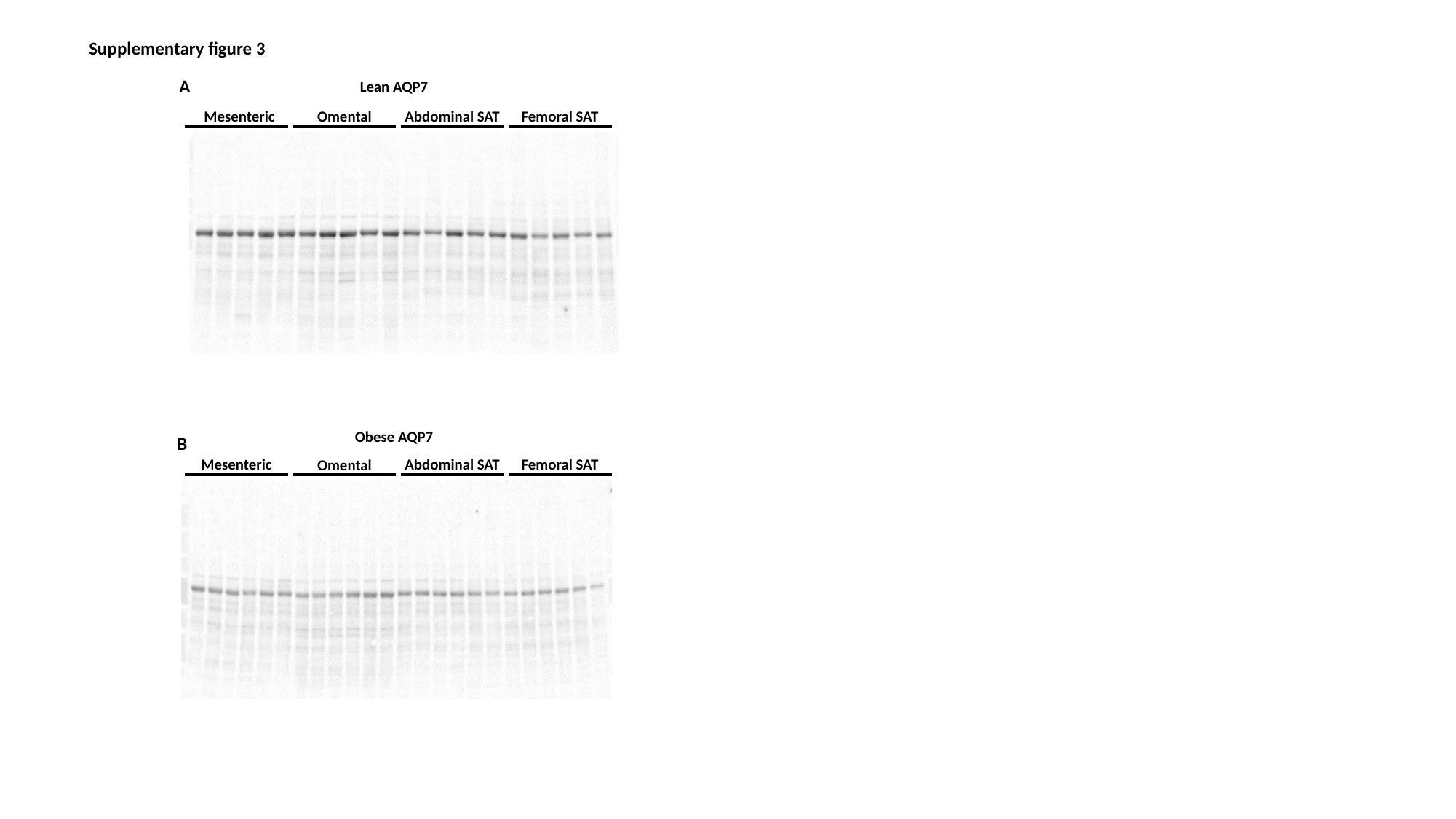

Supplementary figure 3
A
Lean AQP7
Mesenteric
Abdominal SAT
Femoral SAT
Omental
Obese AQP7
B
Mesenteric
Abdominal SAT
Femoral SAT
Omental

## Slide 4
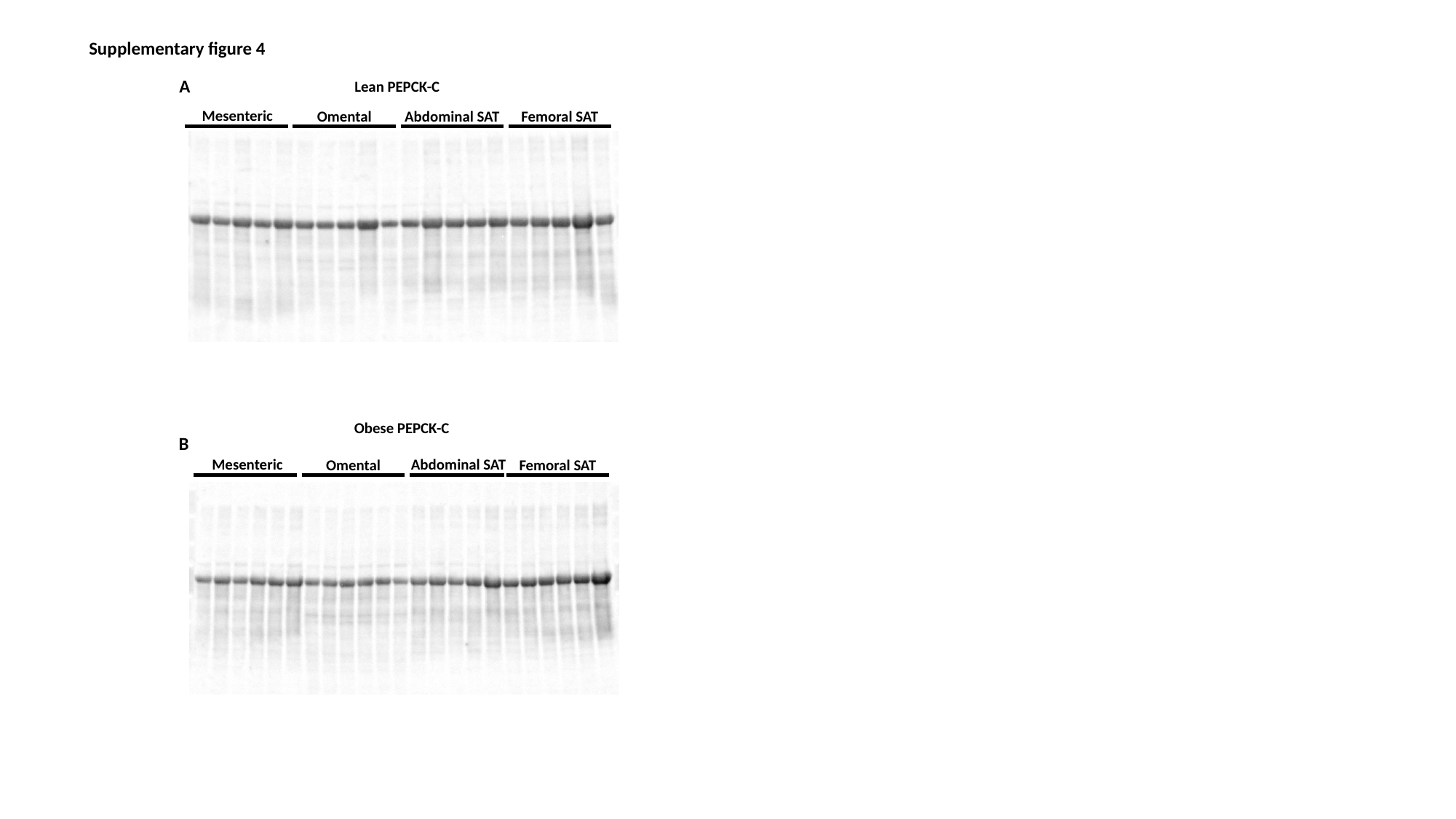

Supplementary figure 4
A
Lean PEPCK-C
Mesenteric
Abdominal SAT
Femoral SAT
Omental
Obese PEPCK-C
B
Mesenteric
Abdominal SAT
Femoral SAT
Omental
